# Synthesis and Biological Evaluation of Homogeneous Thiol‐Linked NHC*‐Au‐Albumin and ‐Trastuzumab Bioconjugates

**DOI:** 10.1002/chem.201800872

**Published:** 2018-05-22

**Authors:** Maria J. Matos, Carlos Labão‐Almeida, Claire Sayers, Oyinlola Dada, Matthias Tacke, Gonçalo J. L. Bernardes

**Affiliations:** ^1^ Department of Chemistry University of Cambridge Lensfield Road Cambridge CB2 1EW United Kingdom; ^2^ Instituto de Medicina Molecular Faculdade de Medicina Universidade de Lisboa Avenida Professor Egas Moniz 1649-028 Lisboa Portugal; ^3^ Albumedix Ltd. Castle Court, 59 Castle Boulevar Nottingham NG7 1FD United Kingdom; ^4^ School of Chemistry University College Dublin Belfield Dublin 4 Ireland

**Keywords:** albumin, anticancer drug, drug delivery, gold(I) complex, targeted therapeutics

## Abstract

Targeted delivery of potent cytotoxic drugs to cancer cells minimizes systemic toxicity and several side effects. NHC*−Au−Cl has already been proven to be a potent anticancer agent. In this study, we explore a strategy based on chemoselective cysteine conjugation of NHC*−Au−Cl to albumin and trastuzumab (Thiomab LC‐V205C) to potentiate drug‐ligand ratio, pharmacokinetics, as well as drug efficacy and safety. This strategy is a step forward towards the use of gold‐based anticancer agents as targeted therapies.

The use of elemental “medicinal gold” by applying gold powder to skin ulcers or “potable gold” containing colloidal gold against infections and inflammations has its origin in the ancient imperial Chinese culture as early as 2500 BC.^1^ In Europe this knowledge was used by alchemists like Paracelsus up to the late‐medieval times. Modern use of gold‐based drugs started in 1890 with the German physician and bacteriologist Robert Koch, who discovered the anti‐tubercular activity of potassium dicyanoaurate in vitro, which unfortunately does not persist in vivo.^2^ There was more success in 1929, when the French physician Jacques Forestier used sodium aurothiopropanol sulfonate to successfully treat rheumatoid arthritis.^3^ These findings led to the development of Auranofin (triethylphosphino gold(I) 2,3,4,6‐tetra‐*O*‐acetyl‐β‐d‐glucopyranosyl‐1‐thiolate, Figure 1), which was introduced into the clinic in 1985 and is used to treat certain cases of arthritis.^4^ Nowadays, repurposing of Auranofin towards cancer and chemical modifications of the stabilising ligands of Au^I^ have led to a large variety of phosphine‐ and even more stable carbene‐gold(I) species with anticancer activity.^5^, ^6^, ^7^, ^8^, ^9^ Some lead compounds developed by Tacke include 1,3‐dibenzyl‐4,5‐diphenyl‐imidazol‐2‐ylidene gold(I) chloride (NHC*−Au−Cl, Figure 1) and its 2′,3′,4′,6′‐tetra‐*O*‐acetyl‐β‐d‐glucopyranosyl‐1′‐thiolate derivative (NHC*−Au−SR, Figure 1). These exhibit average GI_50_ values of 1.78 and 1.95 μm on the NCI 60 cancer cell panel and induce apoptosis through thioredoxin reductase (TrxR) inhibition with IC_50_ values of 1.5 μm for and 3.1 μm; both compounds produce identical promising T/C values of 0.47, when tested against xenografted CAKI‐1 tumours in mice.^10^, ^11^

**Figure 1:**
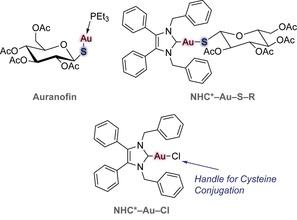
Chemical structures of Auranofin, NHC*−Au−SR and NHC*−Au−Cl. Auranofin and NHC*−Au−SR, especially their thiol‐gold linkages, were the inspiration for the rational design of this project.

Previous cellular uptake studies showed that the presence of the NHC fragment in a gold complex is important for the accumulation of both the metal and the ligand.^12^, ^13^ In addition, a number of strategies explored the attachment of a targeting ligand (i.e., sugars and peptides) through S−Au^I^ bonds. However, functionalisation of the complex with a directing ligand has often led to a decrease in activity.^14^, ^15^, ^16^ A number of reviews summarizing the role of NHC−Au and S−Au conjugates in biomedicine, in particular as anticancer agents, have been published.^17^, ^18^, ^19^, ^20^

This study shows a versatile way to synthesise NHC*−Au−S‐protein bioconjugates by cysteine‐selective gold protein metallation. This strategy was demonstrated by the site‐selective conjugation of NHC*−Au−Cl to albumin and the antibody trastuzumab with the aim of enhancing stability and blood circulation half‐time as well as biodistribution to cancer tissues of the gold‐based anticancer drug.

NHC*−Au−Cl bioconjugation with rHSA: Human serum albumin (HSA) is the most abundant protein in the blood (30–50 g L^−1^ human serum), displaying an important role as transporter. It has multiple ligand binding sites, and a long circulatory half‐life, particularly due to interaction with the recycling neonatal Fc receptor (FcRn). HSA has a reactive free cysteine that proved to be accessible for successful conjugation, resulting in bioconjugates improved serum stability.^21^ These unique characteristics promote HSA as an attractive carrier for delivery and half‐life expansion of drugs. Importantly, it has been shown that 20 % of an injected dose of a radio‐labelled albumin accumulates in rats bearing tumours (5 % of the total body weight), after 24 h,^22^ suggesting that rHSA could be used for cancer targeted drug‐delivery. Furthermore, the adducts of ruthenium‐based drugs such as CORM‐3^23^, ^24^ and NAMI‐A,^25^ formed with albumin in plasma, have been shown to be the active forms of these drugs in vivo, implying the potential of protein conjugation to improve the properties metal‐based drugs.

In this study, we used a recombinant form of human albumin—Recombumin (rHSA, Albumedix Ltd.) to demonstrate the potential of thiol‐gold linked protein‐metal complexes for cancer therapeutics. Using a single equivalent of NHC*−Au−Cl, in NaP_i_ (50 mm, pH 7.0), for 2 h at 37 °C, we observed full conversion to the corresponding thiol‐gold adduct (analysed by LC‐MS, Figure 2 b). We performed the reaction at different pH values of 8.0 and 9.0, and obtained similar results, with no cross‐reactivity or degradation observed. In addition, when the number of equivalents of NHC*−Au−Cl was increased to 5, 10 or 50, selectivity for the single cysteine residue at position 34 on albumin was always observed, which demonstrates the chemoselectivity for cysteine even in the presence of other nucleophilic amino acid side‐chains (e.g., lysine residues). A drug:albumin ratio of 1 was found for all the tested conditions. Our data is in good agreement with literature reports by Ott and co‐workers that showed the quantitative binding of NHC*−Au−Cl complexes to serum proteins.^12^ The formation of Au‐serum protein adducts decreased both gold uptake and activity. To demonstrate that rHSA could act as a carrier for targeting NHC−Au complexes to tumours,^22^ we decided to study the stability of NHC*−Au−rHSA in human plasma and its binding affinity to the neonatal FcRn receptor.

**Figure 2:**
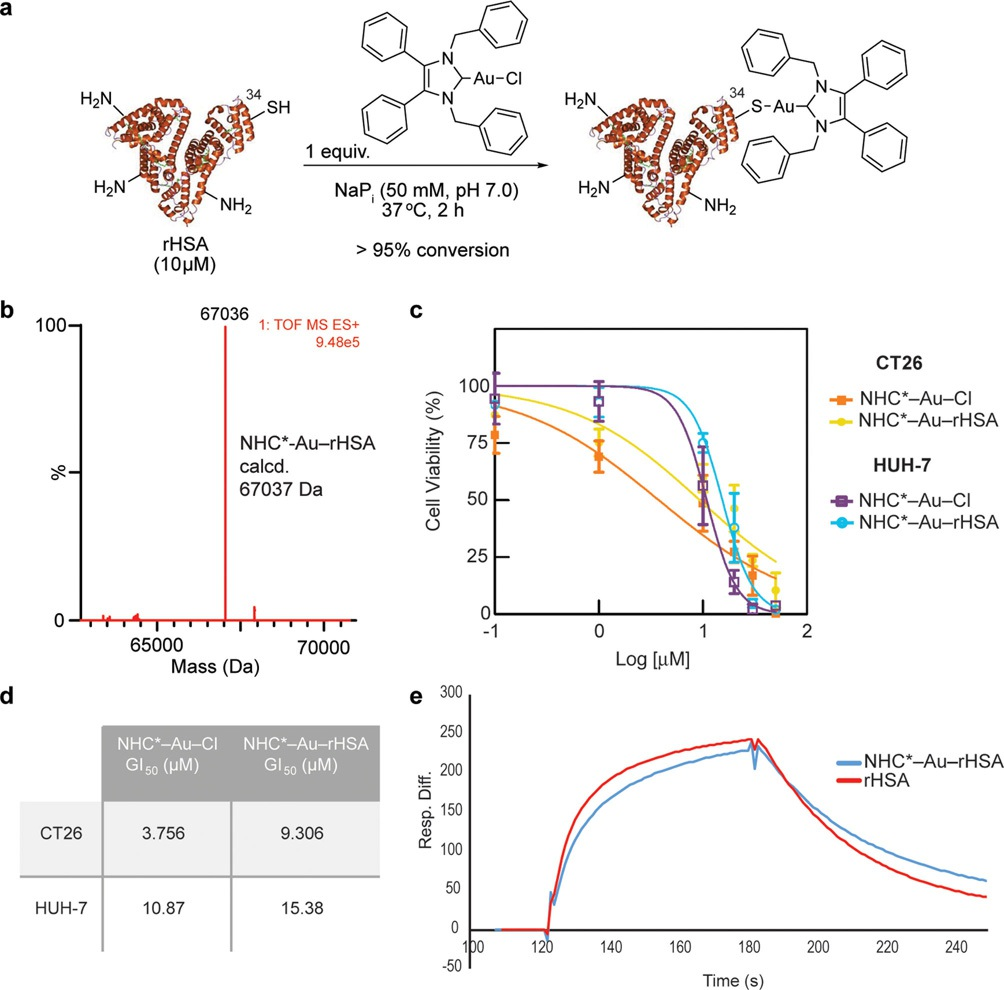
Bioconjugation of rHSA. a) General reaction of rHSA with the NHC*−Au−Cl. b) Electrospray ionization‐MS spectra of NHC*−Au−rHSA (deconvoluted spectra; expected increasing of 596 mass units). c) CT26 and HUH‐7 cells viability after treatment with NHC*−Au−Cl and NHC*−Au−rHSA. Results are shown as a percentage of control (medium+vehicle–PBS) and correspond to 3 biological replicates (mean±s.d.). d) GI_50_ of NHC*−Au−Cl and NHC*−Au−rHSA in CT26 and HUH‐7 cells. e) Surface plasma resonance (SPR) to determine the ability to bind to the FcRn receptor. *K*_D_ for non‐modified rHSA and bioconjugate NHC*−Au−rHSA.

Stability, binding properties and cell viability of NHC*−Au−rHSA: We next evaluated the stability of the NHC*−Au−rHSA in physiological conditions. NHC*−Au−rHSA proved to be stable up to 48 h in human plasma (Figure 12 in the Supporting Information), as observed by LC‐MS analysis. This is an important consideration for protein conjugates that are expected to be administered intravenously and remain stable in circulation. Moreover, analysing surface plasma resonance (SPR) results, we confirmed that NHC*−Au−rHSA retains its ability to bind to the neonatal FcRn receptor: *K*_D_ of rHSA is 2.69 μm and *K*_D_ of NHC*−Au−rHSA is 2.52 μm (Table 1 and Figure 2 e).^26^

**Table 1:** Kinetic analysis at pH 5.5 of rHSA derivatives binding to human FcRn receptor.

Sample	*k*_on_ [10^−3^ Ms^−1^]	*k*_off_ [10^3^ s^−1^]	*K*_D_ [μm]
rHSA	10.42	28.30	2.69
NHC*−Au−rHSA	8.76	22.10	2.52

The sensorgrams (10 μm) show that although the overall *K*_D_ values of the two samples are in good agreement, there are some subtle differences in the “on” and “off” rates, with the native sample (rHSA) having a slightly faster “on” rate and the modified sample (NHC*‐Au‐rHSA) having a slightly slower “off” rate. The main conclusion of the experiment is that the FcRn binding has been retained after conjugation. This data corroborates the potential of our new method to generate stable and functional protein conjugates for in vivo applications. Importantly, this homogeneous NHC*−Au−rHSA bearing a defined drug:albumin ratio of 1 shows an important maintenance in cell‐killing activity because it exhibits comparable cytotoxicity to the free drug (NHC*−Au−Cl), as assessed by CellTiter Blue assay in both CT26 (colon carcinoma) and HUH‐7 (hepatocarcinoma) cancer cells (Figure 2 c,d).

NHC*−Au−Cl bioconjugation with Thiomab LC‐V205C: Traditional non‐surgical methods for cancer treatment (chemotherapy and radiotherapy) possess some fundamental limitations. Specific accumulation in the tumour tissue is still one of the biggest challenges of anticancer therapies. Antibodies are able to recognise antigens overexpressed in cancer cells.^27^ Therefore, there is no surprise that they have been intensively studied as carriers for drugs and contrast agents for cancer therapy and imaging in the last few years.^28^, ^29^ However, the outcome of these efforts is rather modest. It was suggested that the therapeutic index and safety of antibody–drug conjugates (ADCs) can still be significantly improved by using modern site‐selective methods of protein modifications to create next‐generation ADCs with a defined drug:antibody ratio (DAR) at specific sites.^30^ In this study, we used an engineered trastuzumab presenting an additional free cysteine per light chain—Thiomab LC‐V205C (Genentech, Inc.). Using 5 equivalents per light chain of NHC*−Au−Cl, in NaP_i_ (50 mm, pH 7.0), for 2 h at 37 °C, we observed complete conversion to the corresponding thiol‐gold adduct (analysed by LC‐MS, Figure 3 b). We were delighted to observe selectivity for a single cysteine residue per light chain, without any detectable modification in the heavy chain (Figure 3 b).

**Figure 3:**
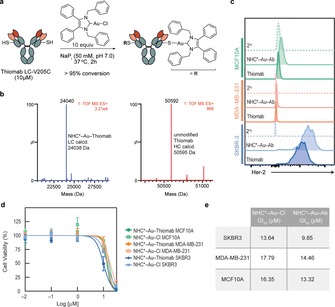
Bioconjugation of Thiomab LC‐V205C. a) General reaction of Thiomab LC‐V205C with the NHC*−Au−Cl. b) Electrospray ionization‐MS spectra of NHC*‐Au‐Thiomab (deconvoluted spectra) for the light (blue) and heavy chain (red)—expected increasing of 596 mass units per light chain; no modifications were observed to the heavy chain mass, as expected. The DAR of this ADC is 2. c) NHC*‐Au‐Thiomab (here referred to as NHC*−Au−Ab) binding affinity in HER2‐overexpressing SKBR3 breast cancer cells, triple‐negative breast cancer cell line MDA‐MB‐231, and non‐tumourogenic breast cells MCF10A by flow cytometry. Naked Thiomab was used as a positive control and a secondary antibody was used as a negative control. d) Viability of MCF‐10A, MDA‐MB‐231 and SKBR3 cells after treatment with NHC*‐Au‐Thiomab or NHC*−Au−Cl for 24 h. Results are shown as a percentage of control (medium+vehicle–PBS) and correspond to 3 biological replicates (mean±s.d.). e) Table with GI_50_ values of NHC*−Au−Cl and NHC*‐Au‐ Thiomab in MCF10A, MDA‐MB‐231 and SKBR3 cells.

We confirmed by flow cytometry that this homogeneous ADC bearing a defined DAR of 2 retained its specificity and capacity to bind to the HER2 antigen in SKBR3 breast cancer cells that overexpress HER2 in comparison with triple negative breast cancer cells, MDA‐MB‐231 and non‐malignant human breast, MCF10A, which do not express HER2 receptors (Figure 3 c). Additionally, we observed that antibody conjugation moderately enhanced the antiproliferative activity of the NHC*−Au−Cl drug as assessed by CellTiter Blue assay (Figure 3 d,e). Importantly, we also observed that the modified antibody displayed higher toxicity in the HER2‐overexpressing SKBR3 cells when compared to triple‐negative breast cancer cells and non‐tumourogenic breast cells, as expected (Figure 3 d,e). With these experiments, we provide evidence of the potential of this site‐selective thiol‐gold conjugation method for the construction of stable and homogeneous ADCs. In addition, this work highlights the potential of directly conjugating drugs that are outside the traditional choices for ADC construction to improve their anticancer profile.

In conclusion, we developed a methodology that allows us to synthesise NHC*−Au−S‐protein bioconjugates using rHSA and a therapeutically used antibody (trastuzumab). A one‐step and irreversible cysteine selective bioconjugation is reported. The reaction can proceed even when using equimolar amounts of the gold‐complex drug, under physiological‐like conditions. The new conjugate, with NHC*−Au−Cl directly linked to the cysteine residue of rHSA, proved to be stable in plasma, and importantly, the protein retained its binding to the FcRn receptor after conjugation. NHC*−Au−rHSA conjugate should, in principle, promote an enhanced stability, half‐time circulation in blood and biodistribution of the gold anticancer drug. This methodology holds great promise and can be applicable to ligands of diverse metal families. In fact, a stable and homogeneous ADC (NHC*‐Au‐Thiomab) was also prepared using the same strategy. The binding of the antibody was maintained and the antiproliferative activity of the metal‐complex drug moderately improved when delivered in the form of a homogenous gold thiol‐linked Thiomab conjugate. This strategy potentiates the uptake of the drug into cancer cells, to reduce unwanted side effects of traditional cancer treatments. Together, these findings mean a step forward for the use of gold‐based anticancer drugs as targeted‐therapies.

## Conflict of interest

The authors declare no conflict of interest.

## Biographical Information

*Dr. Gonçalo Bernardes completed his D.Phil. in 2008 at the University of Oxford. He then undertook postdoctoral work at the Max‐Planck Institute of Colloids and Interfaces, Potsdam, and the ETH Zürich. He was also a Group Leader at Alfama Lda in Portugal. In 2013, he became a Group Leader at the University of Cambridge, where he addresses important questions in life sciences and molecular medicine. He is also the Director of the Chemical Biology and Pharmaceutical Biotechnology Unit at the Instituto de Medicina Molecular, Portugal, a Royal Society University Research Fellow, an Investitagor FCT and the awardee of a Starting Grant from the ERC (TagIt). Dr. Bernardes was the Silver Medal winner (35‐years level) in the 2014 competition of the European Young Chemists Award*.

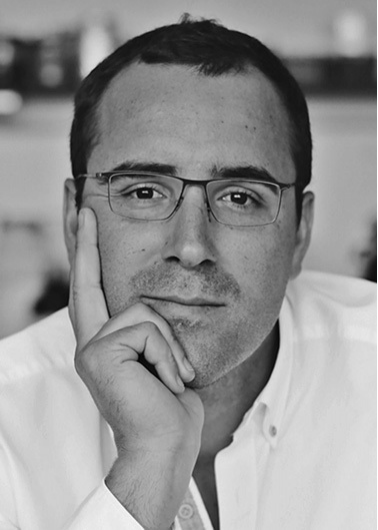

## Supporting information

As a service to our authors and readers, this journal provides supporting information supplied by the authors. Such materials are peer reviewed and may be re‐organized for online delivery, but are not copy‐edited or typeset. Technical support issues arising from supporting information (other than missing files) should be addressed to the authors.

Supplementary
