# Supplementary material for: Synthesis and Biological Evaluation of Homogeneous Thiol‐Linked NHC*‐Au‐Albumin and ‐Trastuzumab Bioconjugates
Source: Chemistry. 2018 May 22;24(47):12250–3. doi: 10.1002/chem.201800872 (PMC6120520; doi:10.1002/chem.201800872)
Supplement: Supplementary file 1 — Supplementary [file CHEM-24-12250-s001.pdf]

CHEMISTRY
A European Journal

Supporting Information

Synthesis and Biological Evaluation of Homogeneous Thiol-Linked NHC*-Au-Albumin and -Trastuzumab Bioconjugates

Maria J. Matos+,[a] Carlos Labão-Almeida+,[b] Claire Sayers,[c] Oyinlola Dada,[d] Matthias Tacke,[d] and Gonçalo J. L. Bernardes*[a, b]

chem_201800872_sm_miscellaneous_information.pdf

SUPPORTING INFORMATION

General procedures for protein/antibody labelling and characterization of the bioconjugates 2

NHC*-Au-Cl bioconjugation with rHSA 5

Stability of NHC*-Au–rHSA bioconjugation in human plasma 11

Determination of FcRn binding by Surface Plasmon Resonance (SPR) 12

NHC*-Au–rHSA cytotoxicity assays 13

NHC*-Au-Cl bioconjugation with Thiomab LC-V205C 15

NHC*-Au–Thiomab cytotoxicity and binding assays 18

General procedures for protein/antibody labelling and characterization of the bioconjugates

General procedure for protein and antibody conjugation with NHC*-Au-Cl

To an eppendorf tube with NaP_i (50 mM, pH 7.0) and DMF (10% of total volume), an aliquot of a stock solution of protein (final concentration 10 μM) was added. Afterwards, a solution of the NHC*–Au–Cl (1 to 10 equiv.) in DMF was added and the resulting mixture was vortexed for 10 seconds. The reaction was mixed for 2 or 24 h, at 37 ºC. A 10 μL aliquot of each reaction time was analysed by LC–MS and conversion to the expected product was observed.

LC–MS method for analysis of protein conjugation

LC–MS was performed on a Water Acquiry UPLC system equipped with a single quadrupole mass detector using an Acquity UPLC protein BEH C4 column, 300 Å, (1.7 mm, 2.1 × 50 mm). Solvents A, water with 0.01% formic acid and B, 71% acetonitrile, 29% water and 0.075% formic acid were used as the mobile phase at a flow rate of 0.2 $mL \cdot min^{-1}$ from 0-20 min, and 0.05 $mL \cdot min^{-1}$ from 20–30 min. The electrospray source was operated with a capillary voltage of 3.0 kV and a cone voltage of 20 V. Nitrogen was used as the desolvation gas at a total flow of 8 $L \cdot h^{-1}$. Total mass spectra were reconstructed from the ion series using the MaxEnt algorithm preinstalled on MassLynx software (v. 4.1 from Waters) according to the manufacturer's instructions. To obtain the ion series described, the major peak(s) of the chromatogram were selected for integration and further analysis.

UPLC for protein/antibody analysis: deionized water containing 0.1% of formic acid (solvent A) and 71% MeCN, 29% H_2O, 0.075% formic acid (solvent B) are used as mobile phase for gradient elution. The gradient program for the purification of protein/antibody is reported in the following table. *(flow 0.2 mL/min from 0–20 min, flow 0.05 mL/min from 20–30 min).

Time interval (min)	% (vol/vol) solvent B
0	28
12	71.2
13	100
16	100
16.5	28
20*	28

Analysis of protein conjugation by LC–MS

A typical analysis of a conjugation reaction by LC–MS is described below. Briefly, the total ion chromatogram, combined ion series and deconvoluted spectra are measured for the staring material (rHSA). This allows to monitor progress of the conversion of the non-modified protein/antibody to the conjugated protein/antibody. After size exclusion purification, Bradford protein analysis is used to determine the yield of the reaction. Identical analyses were carried out for all the conjugation reactions performed in this work.

Before conjugation

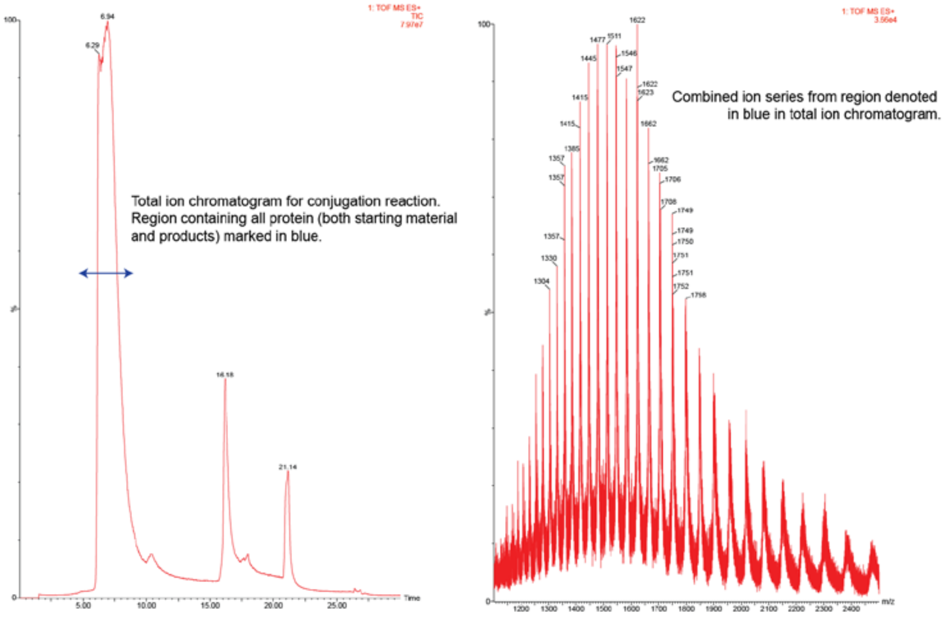

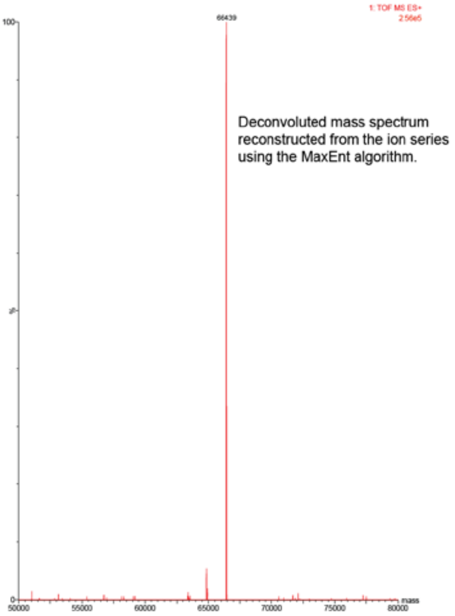

Supporting Figure 1. A typical analysis of a conjugation reaction by LC–MS is described for the rHSA. The total ion chromatogram, combined ion series and deconvoluted spectra are shown. Identical analyses were carried out for all the conjugation reactions performed in this work.

Stability of bioconjugates in human plasma

A 20 µL aliquot of the NHC*–Au–rHSA (10 µM) in NaP_i buffer (50 mM, pH 7.0) was thawed. 1 µL of reconstituted human plasma was added at room temperature and the resulting mixture vortexed for 10 seconds. The resulting reaction mixture was then mixed at 37 ºC. After 24 and 48 h, a 10 µL aliquot of each reaction mixture was analysed by LC–MS.

NHC*–Au–Cl bioconjugation with rHSA

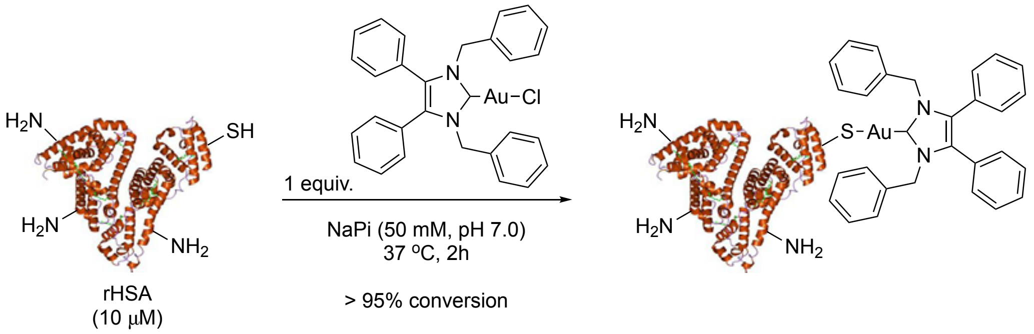

To an eppendorf tube with NaPi (50 mM, pH 7.0, 8.0 or 9.0), DMF (10%), an aliquot of a stock solution of human serum albumin (rHSA, final concentration 10 μM) was added. Afterwards, a solution of NHC*–Au–Cl (1, 5, 10 or 50 equiv.) in DMF was added and the resulting mixture was vortexed for 10 seconds. The reaction was mixed for 2 h at 37 ºC.

Small molecules were removed from the reaction mixture by loading the sample onto a Zeba Spin Desalting Column previously equilibrated with NaP_i (50 mM, pH 7.0).* The sample was eluted via centrifugation (2 min, 1500xg). A 10 μL aliquot was analysed by LC–MS and full conversion to the expected product was observed (calculated mass, 67037 Da; observed mass, 67037 Da).

* These columns are described to have at least 95% retention (removal) of salts and other small molecules (<1000 MW). However, when the reaction was scaled up for *in vitro* studies, this procedure was followed by a dialyses to optimize the efficiency of the method. The sample was dialysed against 0.5 L of NaP_i (50 mM, pH 7.0), stirring, overnight, at room temperature. The buffer solution was changed after 2 h. The following day, the buffer solution was changed once again. After 24 h, the sample was analysed by LC-MS and the concentration determined with a SpectraMax i3x.

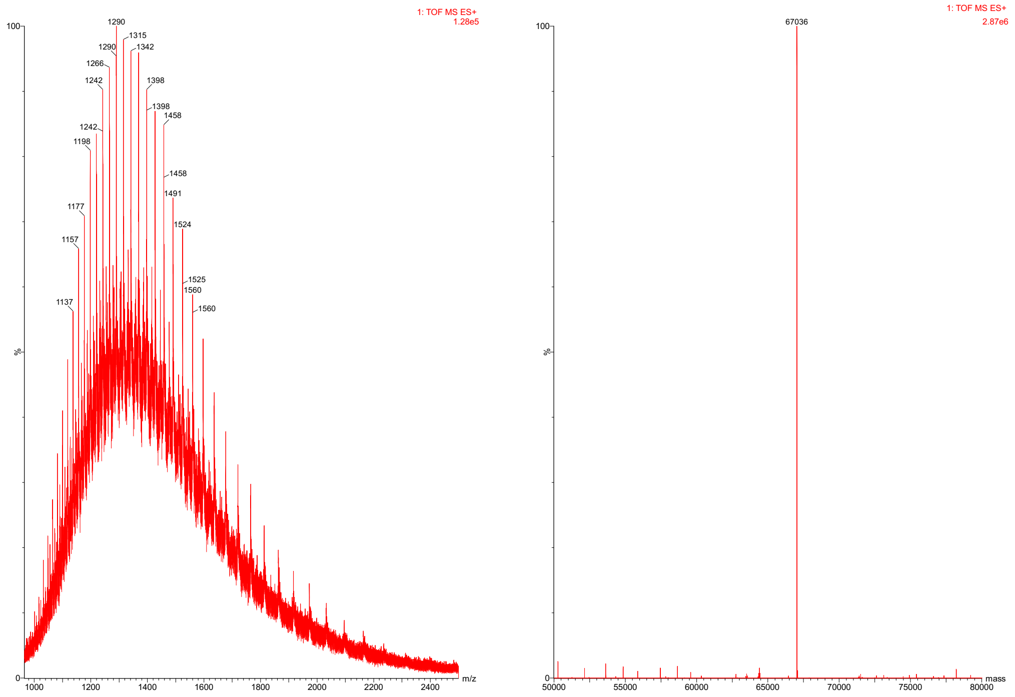

Supporting Figure 2. Combined ion series and deconvoluted mass spectrum of the reaction of rHSA (10 µM) with 1 equiv. of NHC*–Au–Cl, NaP_i (50 mM, pH 7.0) after 2 h at 37 ºC.

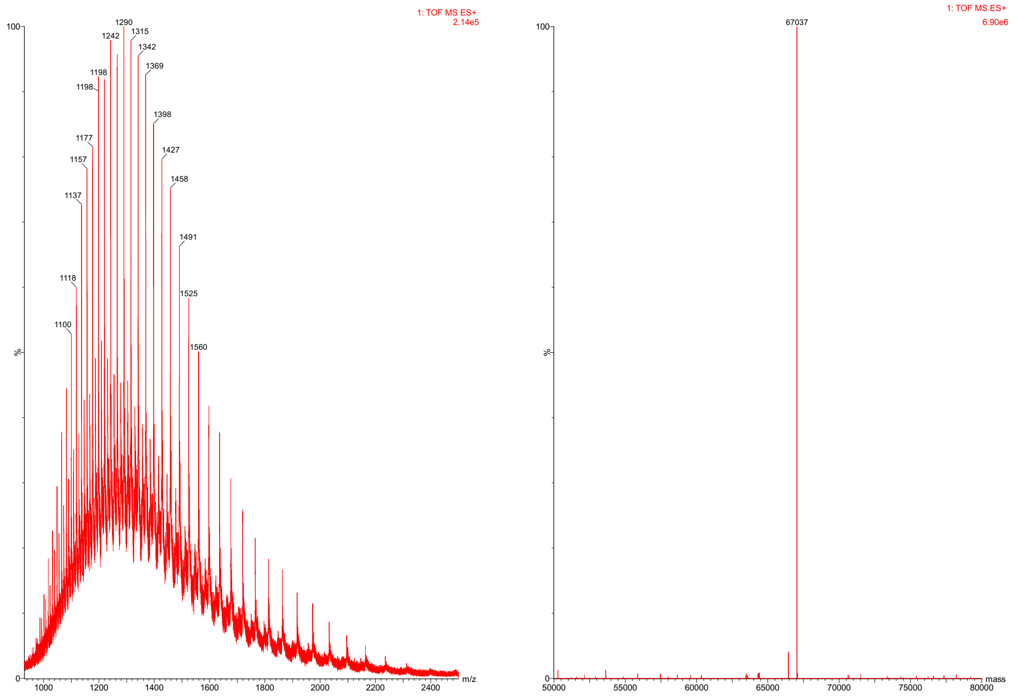

Supporting Figure 3. Combined ion series and deconvoluted mass spectrum of the reaction of rHSA (10 µM) with 5 equiv. of NHC*–Au–Cl, NaP_i (50 mM, pH 7.0) after 2 h at 37 ºC.

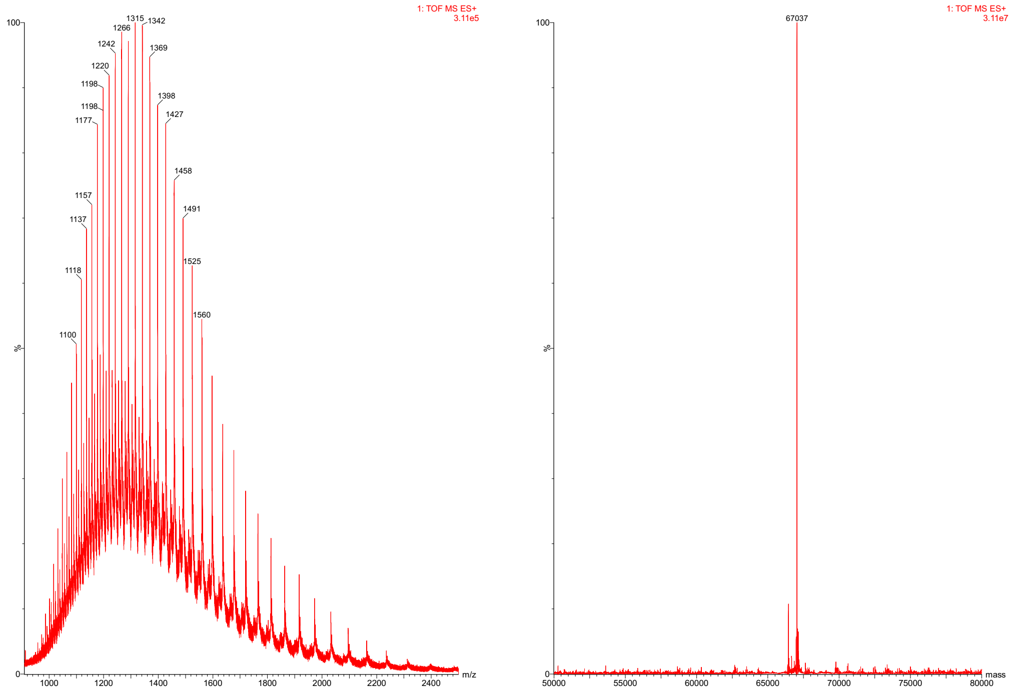

Supporting Figure 4. Combined ion series and deconvoluted mass spectrum of the reaction of rHSA (10 μM) with 10 equiv. of NHC*–Au–Cl, NaP_i (50 mM, pH 7.0) after 2 h at 37 ºC.

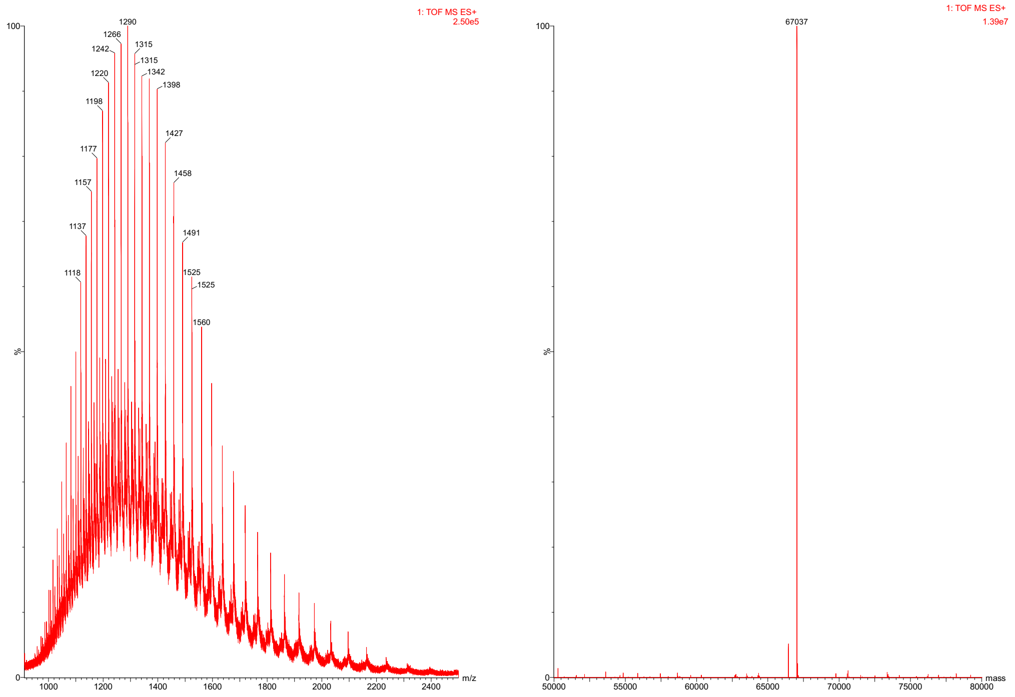

Supporting Figure 5. Combined ion series and deconvoluted mass spectrum of the reaction of rHSA (10 μM) with 50 equiv. of NHC*–Au–Cl, NaP_i (50 mM, pH 7.0) after 2 h at 37 ºC.

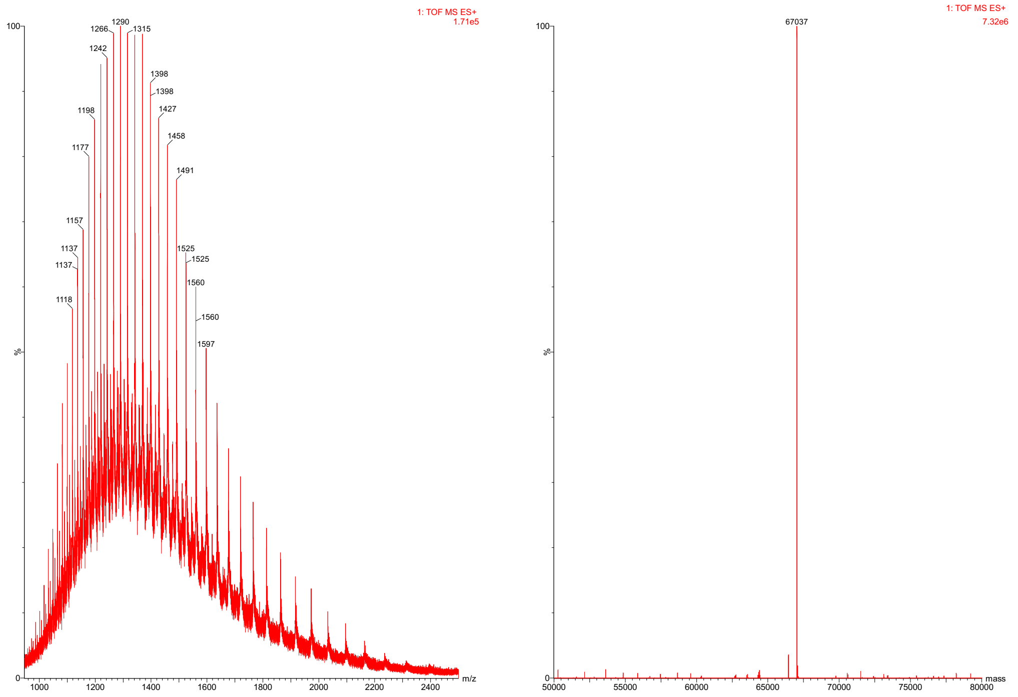

Supporting Figure 6. Combined ion series and deconvoluted mass spectrum of the reaction of rHSA (10 μM) with 1 equiv. of NHC*–Au–Cl, NaP_i (50 mM, pH 8.0) after 2 h at 37 ºC.

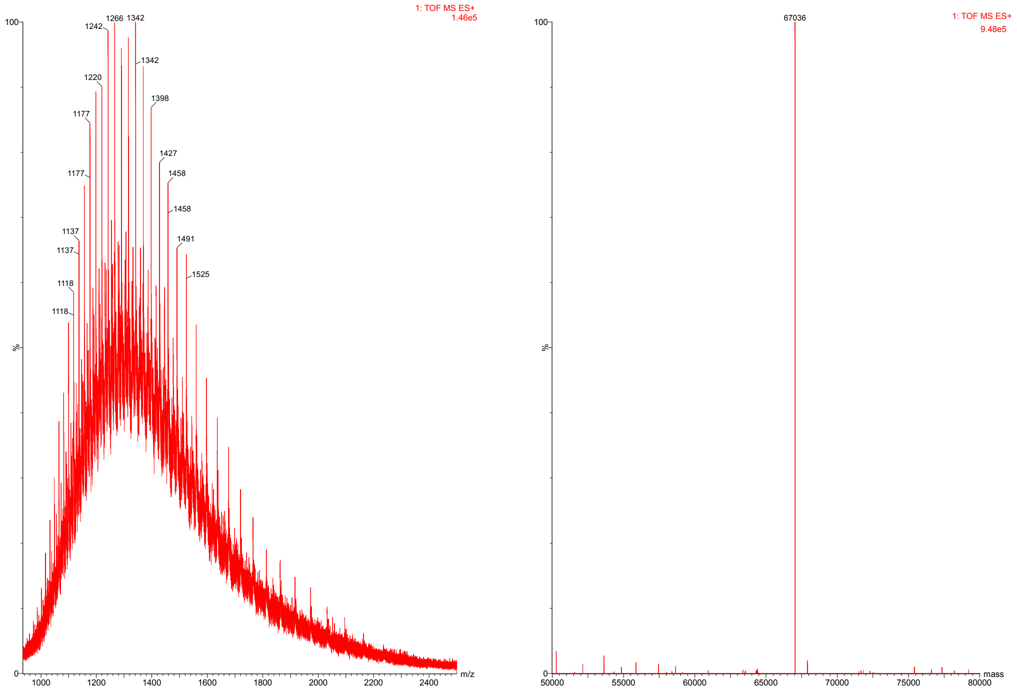

Supporting Figure 7. Combined ion series and deconvoluted mass spectrum of the reaction of rHSA (10 μM) with 5 equiv. of NHC*–Au–Cl, NaP_i (50 mM, pH 8.0) after 2 h at 37 ºC.

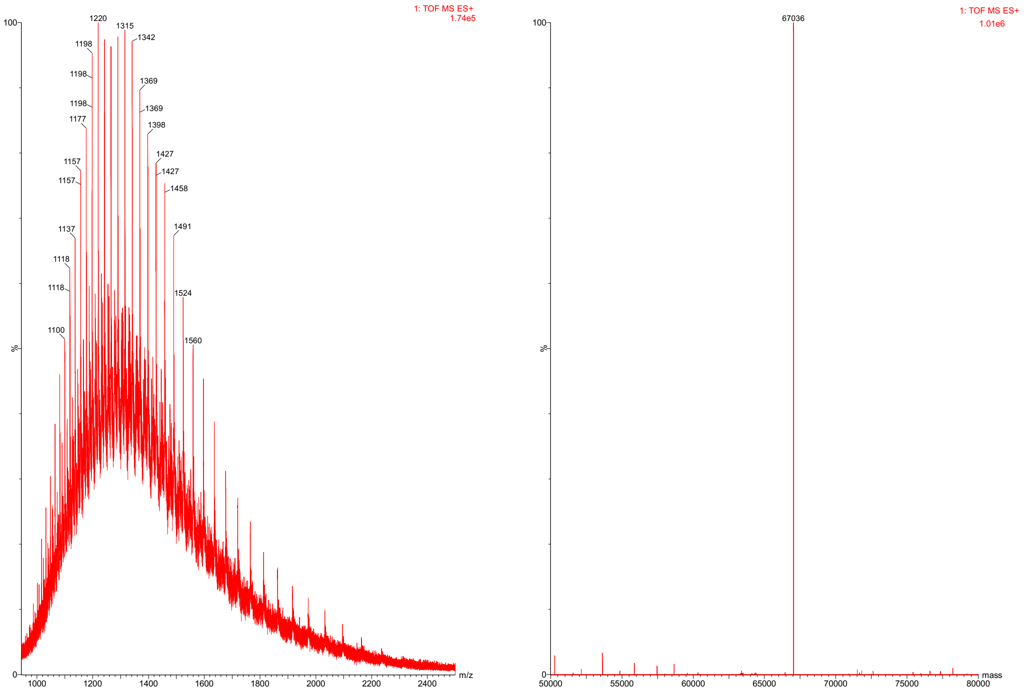

Supporting Figure 8. Combined ion series and deconvoluted mass spectrum of the reaction of rHSA (10 µM) with 10 equiv. of NHC*-Au-Cl, NaP_i (50 mM, pH 8.0) after 2 h at 37 ºC.

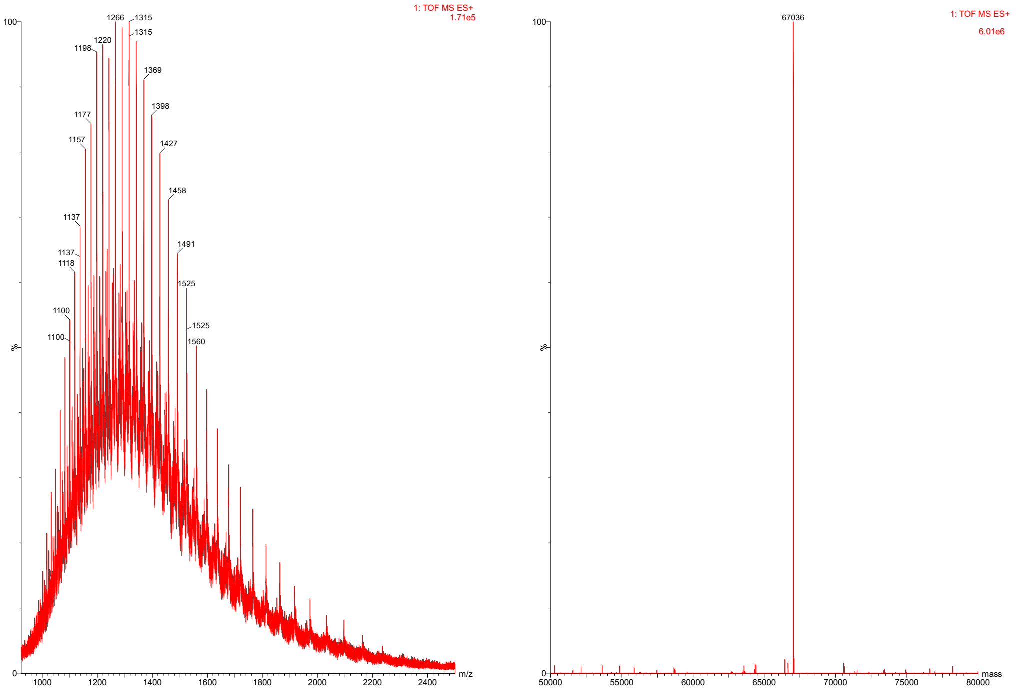

Supporting Figure 9. Combined ion series and deconvoluted mass spectrum of the reaction of rHSA (10 µM) with 50 equiv. of NHC*–Au–Cl, NaP_i (50 mM, pH 8.0) after 2 h at 37 ºC.

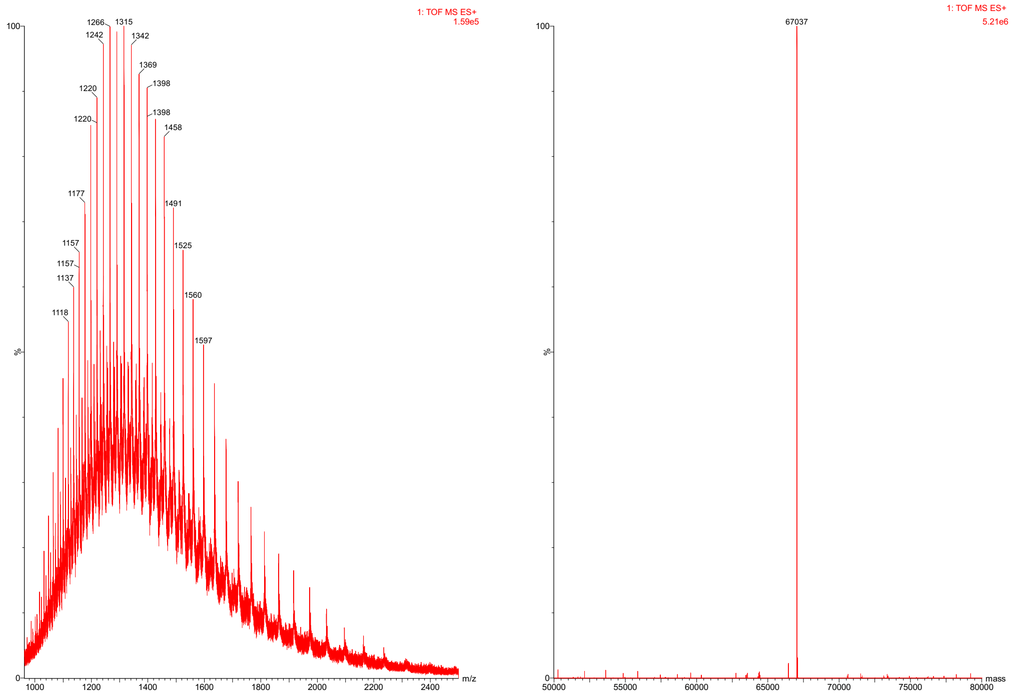

Supporting Figure 10. Combined ion series and deconvoluted mass spectrum of the reaction of rHSA (10 µM) with 1 equiv. of NHC*–Au-Cl, NaP_i (50 mM, pH 9.0) after 2 h at 37 ºC.

Stability of NHC*–Au–rHSA bioconjugation in human plasma

A 20 µL aliquot of NHC*-Au–rHSA (10 µM) in 50 mM NaP_i buffer at pH 7.0 was thawed. 1 µL of reconstituted human plasma (Sigma-Aldrich) was added at room temperature and the resulting mixture vortexed for 10 seconds. The resulting reaction mixture was then mixed at 37 ºC. After 24 and 48 h, a 10 µL aliquot of each reaction mixture was analysed by LC–MS. No significant degradation of the adduct was observed at either time point.

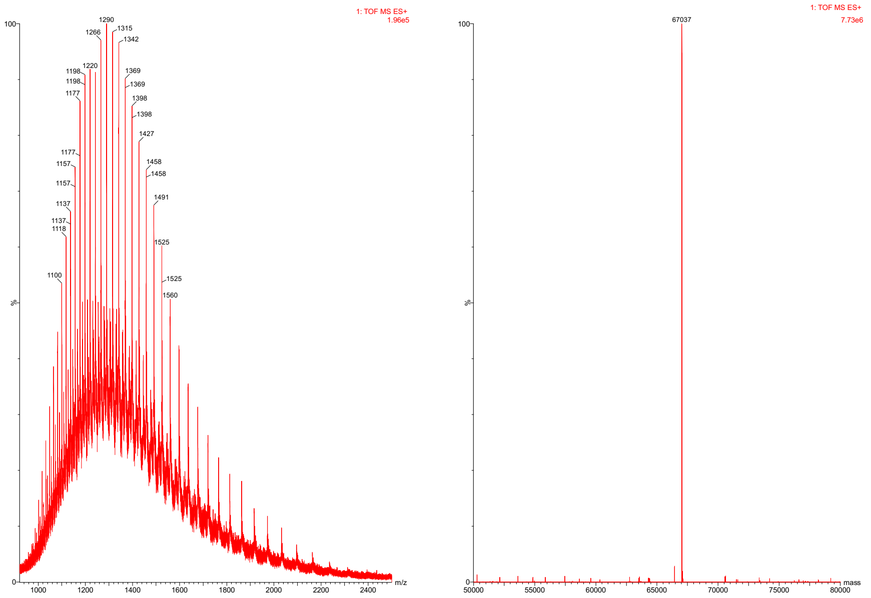

Supporting Figure 11. Combined ion series and deconvoluted mass spectrum of NHC*–Au–rHSA after incubation in human plasma for 24 h at 37 ºC.

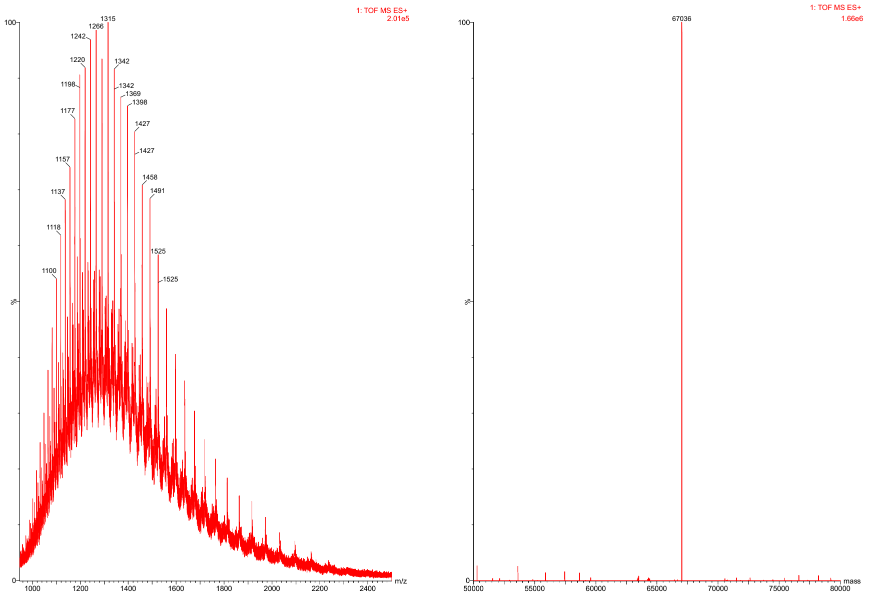

Supporting Figure 12. Combined ion series and deconvoluted mass spectrum of NHC*–Au–rHSA after incubation in human plasma for 48 h at 37 ºC.

Determination of FcRn binding by Surface Plasmon Resonance (SPR)

A Biacore 3000 instrument (GE Healthcare) was used with CM5 sensor chips coupled with mFcRn (cynomolgus monkey FcRn) or hFcRn (1,000 resonance units) using amine coupling chemistry as described by the manufacturer. The coupling was performed by injecting 5–10 μg/mL of each protein into 10 mm sodium acetate, pH 5.0 (GE Healthcare). For all experiments, phosphate buffer (67 mM phosphate buffer, 0.15 M NaCl, 0.005% Tween 20) with pH 5.5, 6.0, or 7.4 was used as running buffer and dilution buffer. Regeneration of the surfaces were achieved using injections of HBS-EP buffer (0.01 M HEPES, 0.15 M NaCl, 3 mM EDTA, 0.005% surfactant P20) at pH 7.4 (GE Healthcare). Kinetic measurements were performed by injecting serial dilutions of 10–0.078 μM of albumin variants with a flow rate of 40 μL/min at 25 °C. For all sensorgrams, data were zero adjusted, and the reference cell value was subtracted. Binding kinetic constants were calculated using the predefined binding models provided by the BIAevaluation 4.1 software.

Sample	k_a	k_d	K_A	K_D
HSA	10.42	28.30	0.37	2.69
NHC*-Au–rHSA	8.76	22.1	0.397	2.52

Concentrations used to calculate the above were 10, 5, 2.5, 1.25. 0.625, 0.313, 0.156 and 0.078 μM.

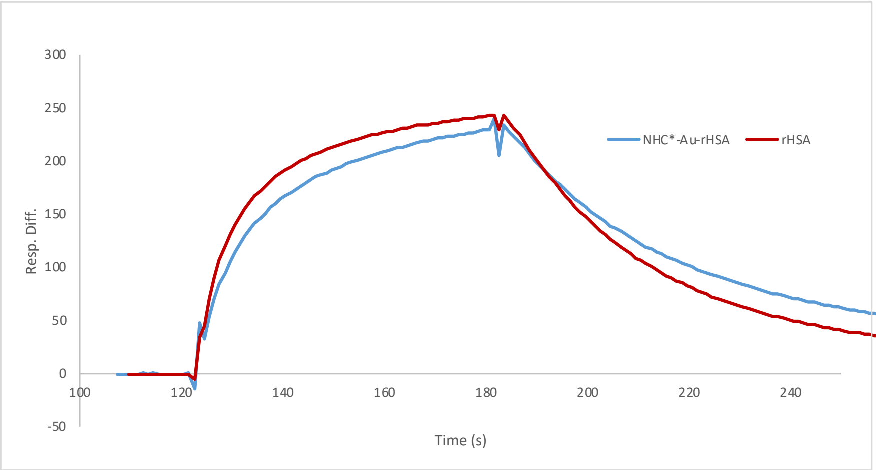

Supporting Figure 13. Assessment of the FcRn binding properties of NHC*–Au–rHSA. Biacore SPR assessment of human FcRn binding of albumins at 10 μM.

NHC*-Au–rHSA cytotoxicity assays

Cell culture

CT26 cells were sourced from Jocelyne Demengeot's research group at IGC (Instituto Gulbenkian de Ciência), Portugal, and HUH-7 cells were kindly gifted by Maria Mota's lab at iMM-JLA (Instituto de Medicina Molecular João Lobo Antunes), Portugal. Cells were cultured in Dulbecco's modified Eagle medium (DMEM), supplemented with 10 mM of HEPES buffer, 1 mM of sodium pyruvate, 100 U/mL of penicillin and 100 μg/mL of Streptomycin and complemented with 1% non-essential amino acids (NEAA), 1% GlutaMAX supplement and 10% heat-inactivated fetal bovine serum (HI FBS). All cells were maintained in a humidified incubator at 37 ºC and 5% CO_2. Every reagent was purchased from Gibco by Life Technologies (Thermo Fisher Scientific) unless stated otherwise.

Viability Assays

Cells were seeded at 10,000 cells per well into flat-bottom 96-well plates. NHC*–Au-Cl and NHC*–Au–rHSA were added after allowing the cells to settle overnight. Relative viability was assessed by using the CellTiter Blue assay (Promega) according to manufacturer's instructions. Fluorescence was determined after 24 h of incubation at 37 ºC on a fluorescent plate reader (TECAN Infinite M200 – excitation/emission 560/590 nm). Obtained values were normalized to the respective controls.

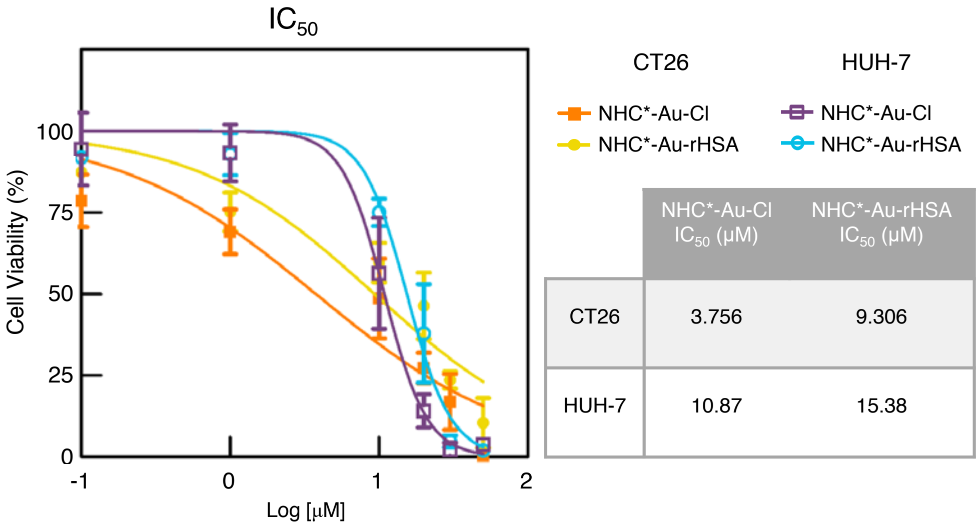

	NHC*-Au-Cl IC$_{50}$ (μM)	NHC*-Au-rHSA IC$_{50}$ (μM)
CT26	3.756	9.306
HUH-7	10.87	15.38

Supporting Figure 14. Concentration-effect curves: cell viability (%) plotted with logarithmic concentration (μM) for both CT26 (colon carcinoma) and HUH-7 (hepatocarcinoma) cancer cells.

NHC*–Au–Cl bioconjugation with Thiomab LC-V205C

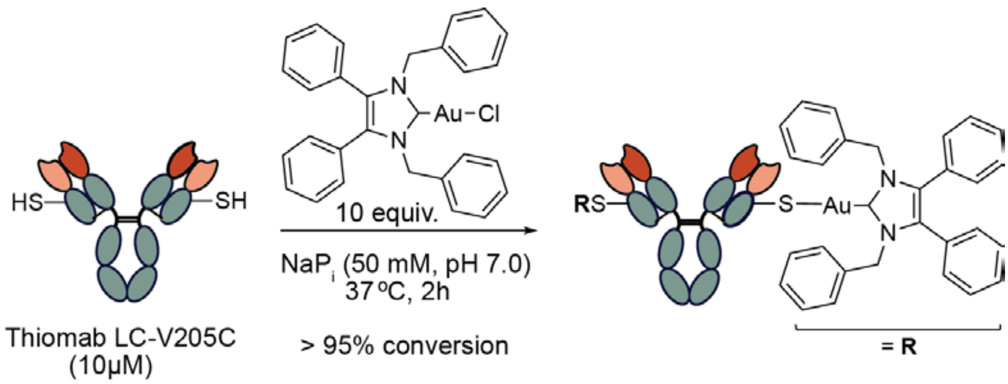

To an eppendorf tube with NaP_i (50 mM, pH 7.0), DMF (10%), an aliquot of a stock solution of Thiomab LC-V205C (final concentration 10 µM) was added. Afterwards, a solution of NHC*–Au-Cl (10 equiv. –5 equiv. per light chain) in DMF was added and the resulting mixture was vortexed for 10 seconds. The reaction was mixed for 2 h at 37 ºC. Small molecules were removed from the reaction mixture by loading the sample onto a Zeba Spin Desalting Column previously equilibrated with NaP_i (50 mM, pH 7.0). The sample was eluted via centrifugation (2 min, 1500xg).* A 10 µL aliquot was analysed by LC–MS and full conversion to the expected product was observed (calculated mass, 24038 Da; observed mass, 24040 Da).

* These columns are described to have at least 95% retention (removal) of salts and other small molecules (<1000 MW). However, when the reaction was scaled up for *in vitro* studies, this procedure was followed by a dialyses to optimize the efficiency of the method. The sample was dialysed against 0.5 L of NaP_i (50 mM, pH 7.0), stirring, overnight, at room temperature. The buffer solution was changed after 2 h. The following day, the buffer solution was changed once again. After 24 h, the sample was analysed by LC-MS and the concentration determined with a SpectraMax i3x.

LC-MS for the starting material (unmodified Thiomab LC-V205C):

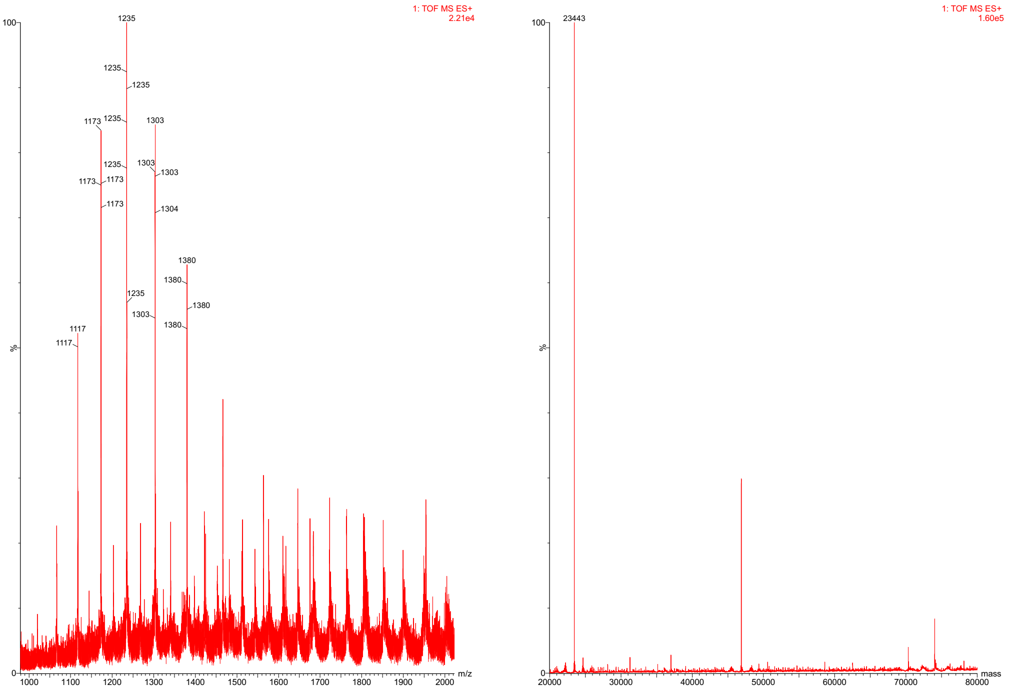

Supporting Figure 15. Combined ion series and deconvoluted mass spectrum of Thiomab LC-V205C (10 µM) – light chain (calculated mass, 23441 Da; observed mass, 23443 Da).

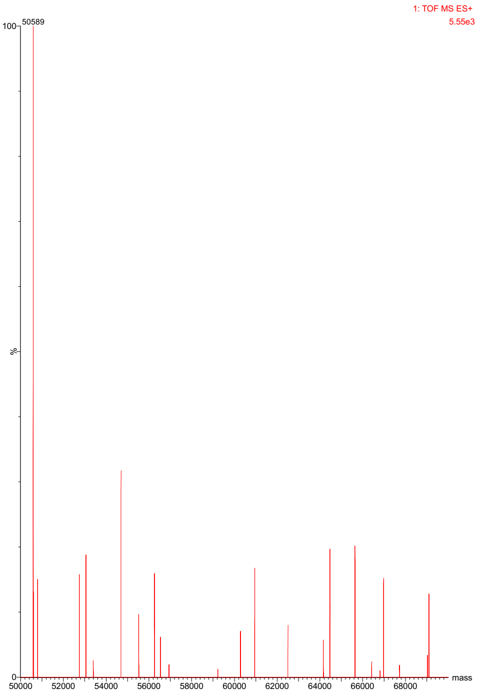

Supporting Figure 16. Combined ion series and deconvoluted mass spectrum of the reaction of Thiomab LC-V205C (10 µM) – heavy chain (calculated mass, 50595 Da; observed mass, 50589 Da).

LC-MS for the reaction (NHC–Au–Thiomab bioconjugate):*

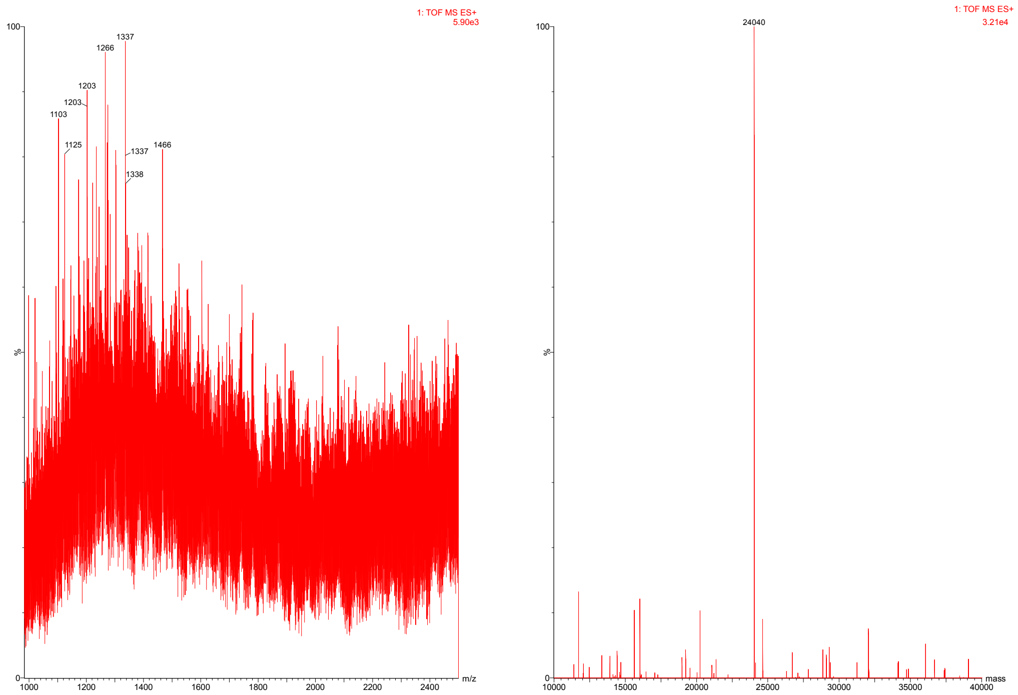

Supporting Figure 17. Combined ion series and deconvoluted mass spectrum of the reaction of Thiomab LC-V205C (10 µM) with 10 equiv. of NHC*-Au-Cl, NaPi (50 mM, pH 7.0) after 2 h at 37 ºC – light chain.

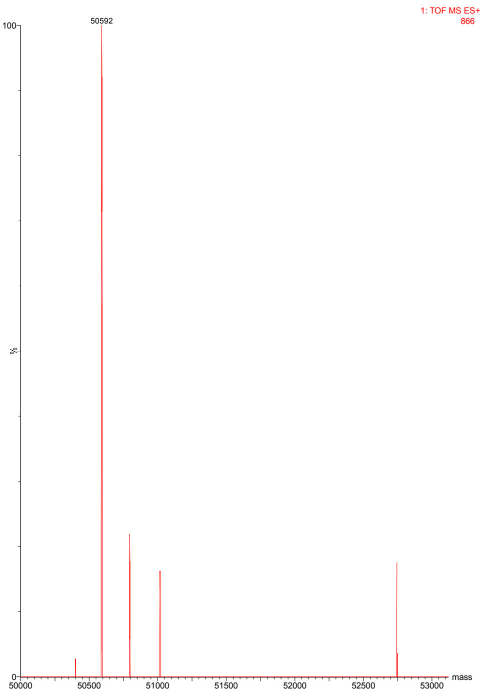

Supporting Figure 18. Deconvoluted mass spectrum of the reaction of Thiomab LC-V205C (10 µM) with 10 equiv. of NHC*–Au-Cl, NaPi (50 mM, pH 7.0) after 2 h at 37 ºC – non-modified heavy chain (calculated mass, 50595 Da; observed mass, 50592 Da).

NHC*–Au–Thiomab cytotoxicity and binding assays

Cell Culture

SKBR3 cell line was kindly provided by Dr. Luís Costa and Dr. Sandra Casimiro and MCF10A cells were a generous offer from CFranco's Lab at Instituto de Medicina Molecular João Lobo Antunes (iMM-JLA), Portugal. MDA-MB-231 cells were purchased from the American Type Culture Collection (ATCC). All reagents were purchased from Gibco by Life Technologies (Thermo Fisher Scientific) unless stated otherwise. All cells with the exception of MCF10A were cultured in Dulbecco's modified Eagle medium (DMEM), supplemented with 10 mM of HEPES buffer, 1 mM of sodium pyruvate, 100 U/mL of penicillin and 100 µg/mL of streptomycin and complemented with 1% non-essential amino acids (NEAA), 1% GlutaMAX supplement and 10% heat-inactivated fetal bovine serum (HI FBS). MCF10A cells were cultured in DMEM/F12 Ham's Mixture supplemented with 10% Equine Serum (Gemini Bio), EGF 20 ng/mL (Peprotech), insulin 10µg/mL (Sigma), hydrocortisone 0.5 mg/mL (Sigma), cholera toxin 100 ng/mL (Sigma), 100 U/mL of penicillin and 100 µg/mL streptomycin. All cells were maintained in a humidified incubator at 37 ºC and 5% CO_2.

Viability Assays

Cells were seeded at 10,000 cells per well into flat-bottom 96-well plate. NHC*–Au–Cl and NHC*–Au–Thiomab were added after allowing the cells to settle overnight. Relative viability was assessed by using the CellTiter Blue assay (Promega) according to manufacturer's instructions. Fluorescence was determined after 24 h of incubation at 37 °C on a fluorescent plate reader (TECAN Infinite M200 – excitation/emission 560/590 nm) and obtained values were normalized to the respective controls.

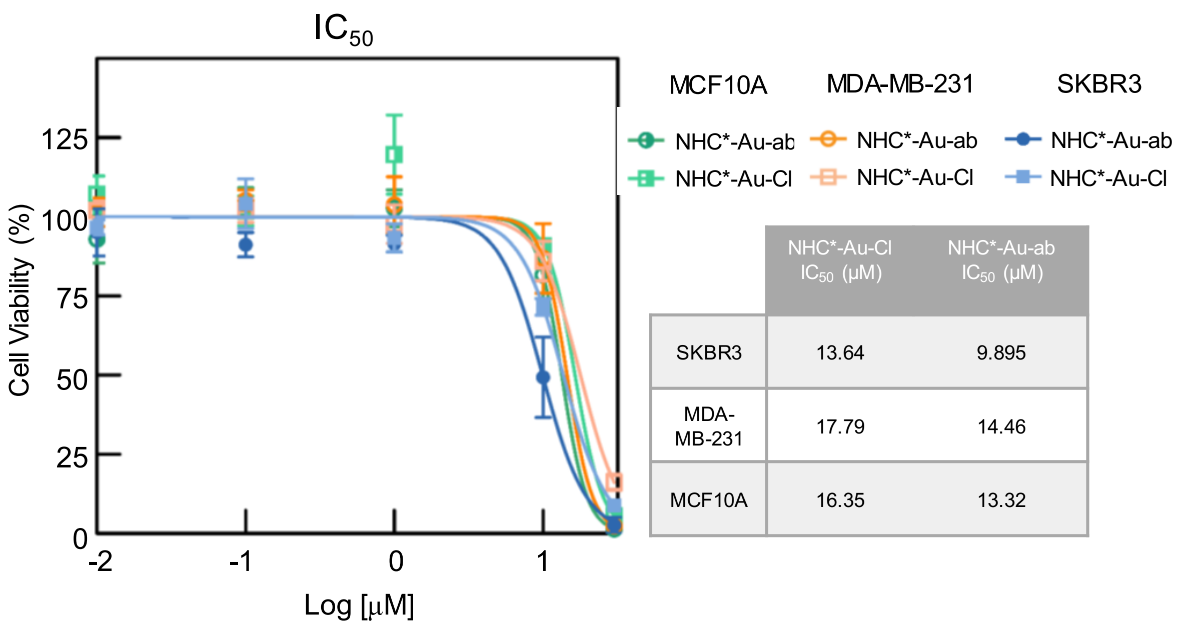

	NHC*-Au-Cl IC_{50} (µM)	NHC*-Au-ab IC_{50} (µM)
SKBR3	13.64	9.895
MDA-MB-231	17.79	14.46
MCF10A	16.35	13.32

Supporting Figure 19. Cell viability after treatment with NHC*–Au–Cl and NHC*–Au–Thiomab assessed by using the CellTiter Blue assay in MCF10A, MDA-MB-231 and SKBR3 breast cancer cells.

Flow Cytometry

Cells were split at confluency with TrypLE Express and collected at 100,000 cells per well into a round-bottom 96-well plate. After centrifuging and removing the culture medium, cells were incubated in culture medium with 100nM of Thiomab or NHC*–Au-Thiomab for 1 h at 4 ºC. Finally, cells were washed and probed with a secondary Alexa Fluor 647 goat anti-human IgG (H+L) (Invitrogen by Thermo Fisher Scientific) for 1 h at 4 ºC, washed again and analysed on a BD LSRFortessa equipped with a FACS Diva Software.

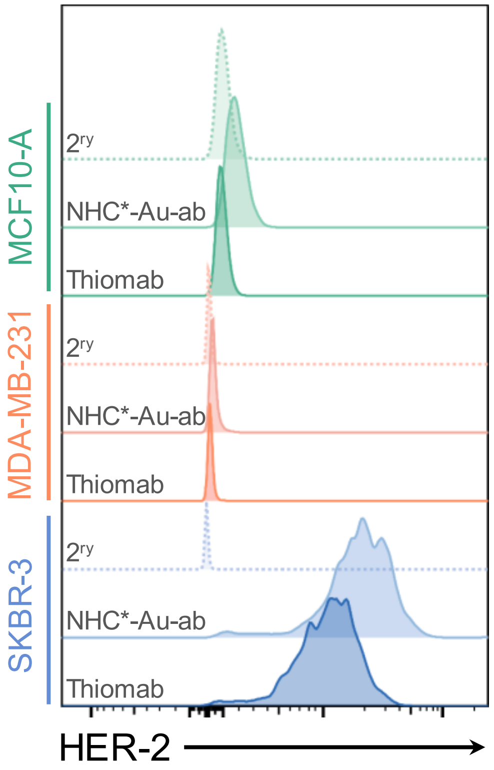

Supporting Figure 20. Binding assay assessed by flow cytometry in SKBR3 breast cancer cells (overexpressing HER2 receptor), triple-negative breast cancer cell line MDA-MB-231, and non-tumorogenic breast cells MCF10A. Thiomab was used as positive control and a secondary antibody was used as negative control.
